# An amputation resets positional information to a proximal identity in the regenerating zebrafish caudal fin

**DOI:** 10.1186/1471-213X-12-24

**Published:** 2012-08-25

**Authors:** Ana Sofia Azevedo, Sara Sousa, António Jacinto, Leonor Saúde

**Affiliations:** 1 Instituto de Medicina Molecular e Instituto de Histologia e Biologia do Desenvolvimento, Faculdade de Medicina da Universidade de Lisboa, Lisbon, 1649-028, Portugal; 2 Instituto Gulbenkian de Ciência, Oeiras, P-2780-156, Portugal; 3 PhD Programme in Experimental Biology and Biomedicine, Centro de Neurociências e Biologia Celular, Universidade de Coimbra, Coimbra, 3004-517, Portugal; 4 CEDOC – Faculdade de Ciências Médicas, Universidade Nova de Lisboa, Lisbon, 1169-056, Portugal

**Keywords:** Caudal fin regeneration, Bony ray bifurcations, Shh, Fgf, Zebrafish

## Abstract

**Background:**

Zebrafish has emerged as a powerful model organism to study the process of regeneration. This teleost fish has the ability to regenerate various tissues and organs like the heart, spinal cord, retina and fins. In this study, we took advantage of the existence of an excellent morphological reference in the zebrafish caudal fin, the bony ray bifurcations, as a model to study positional information upon amputation. We investigated the existence of positional information for bifurcation formation by performing repeated amputations at different proximal-distal places along the fin.

**Results:**

We show that, while amputations performed at a long distance from the bifurcation do not change its final proximal-distal position in the regenerated fin, consecutive amputations done at 1 segment proximal to the bifurcation (near the bifurcation) induce a positional reset and progressively shift its position distally. Furthermore, we investigated the potential role of Shh and Fgf signalling pathways in the determination of the bifurcation position and observed that they do not seem to be involved in this process.

**Conclusions:**

Our results reveal that, an amputation near the bifurcation inhibits the formation of the regenerated bifurcation in the pre-amputation position, inducing a distalization of this structure. This shows that the positional memory for bony ray bifurcations depends on the proximal-distal level of the amputation.

## Background

Tissue regeneration in humans can occur in a limited extent in structures like the skin, gut, skeletal muscle, bone, digit tips, liver and blood. However, other vertebrate species have the extraordinary capacity to regenerate lost tissues and organs throughout adult life. One of such organisms is the zebrafish, a well-established model to study general mechanisms of regeneration, since it is able to regenerate fins, scales, retina, spinal cord and heart among other internal organs [1].

Due to its accessibility, caudal fin regeneration is an example of a powerful and efficient adult model for regenerative studies. The zebrafish caudal fin is composed of several segmented bony rays, mesenchymal tissue, blood vessels and nerve axons. Each bony ray is made of two concave hemirays and, with the exception of the most lateral rays, is bifurcated in a distal position within the fin [2]. These bifurcations are responsible for generating the characteristic shape of the caudal fin.

In the zebrafish caudal fin, an amputation triggers a regenerative program that occurs in three phases: wound healing, blastema formation and regenerative outgrowth. Within the first 12 hour-post-amputation (hpa), the injury is healed through migration of epidermal cells that cover and close the wound [2]. In the next 12–48 hpa, the wound epithelium thickens forming an apical epidermal cap (AEC) and the tissue proximal to the amputation plane disorganizes, begins to proliferate and migrates distally to form the blastema, which is a mass of proliferating cells [2]. The onset of regenerative outgrowth starts at 48 hpa, and at this stage the blastema becomes subdivided into a distal region comprising slow proliferative cells and an intensely proliferative proximal region [3]. Within 2 weeks after amputation, the blastema reconstitutes the original architecture of the caudal fin with all its different tissues and structures [3].

Although we are beginning to understand the molecular mechanisms of regeneration, it is becoming clear that distinct pathways are activated upon amputation. Fibroblast growth factor (Fgf) signalling seems to be required for blastema formation [4], canonical Wnt/β-catenin signalling enhances proliferation of progenitors cells while non-canonical Wnt/Planar cell polarity (PCP) pathway seems to promote the opposite [5] and Hedgehog (Hh) signalling seems to play a latter role by controlling differentiation into bone [6]. A tight control of cell proliferation and differentiation is critical to regenerate a fully functional caudal fin. Nonetheless, equally important is to be able to reconstitute the relative arrangement of the different regenerating tissues and structures, which means that during fin regeneration there must be ways of keeping positional memory. This is a fascinating question in the regeneration field for which we know very little.

In the present study, we took advantage of the zebrafish caudal fin as a model to study positional information of bony ray bifurcations upon amputation, since the stereotypic proximal-distal position of these bifurcations provides an excellent morphological reference. We tested how positional information of bony ray bifurcations is affected by repeated amputations performed at different levels along the proximal-distal axis of the fin. We show that there is a progressive distalization of the position of the bifurcations in the regenerated fin, when the repeated amputations were done proximally near the bifurcation. On the other hand, after a first amputation, its position is maintained in subsequent amputations done near the base of the fin. Thus, we show for the first time that the positional memory of the bifurcation is maintained in proximal but not in distal amputations.

## Results

### Repeated amputations progressively shift the bifurcation position distally

We have previously described an amputation protocol that allowed us to conclude that the regenerative capacity of the zebrafish caudal fin is not affected by repeated amputations or ageing [7]. In this protocol, the caudal fin was subjected to three amputations every month and this was repeated 10 times. During the first 6 months (corresponding to the first 15 cuts) the third consecutive amputation (the last before allowing the fin to completely regenerate) was done three segments below the most proximal bony ray bifurcation (near the bifurcation). In the following 4 months (corresponding to the next 12 cuts), the third consecutive amputation was done 4 segments distally from the base of the fin (near the base of the fin).

**Figure 1:**
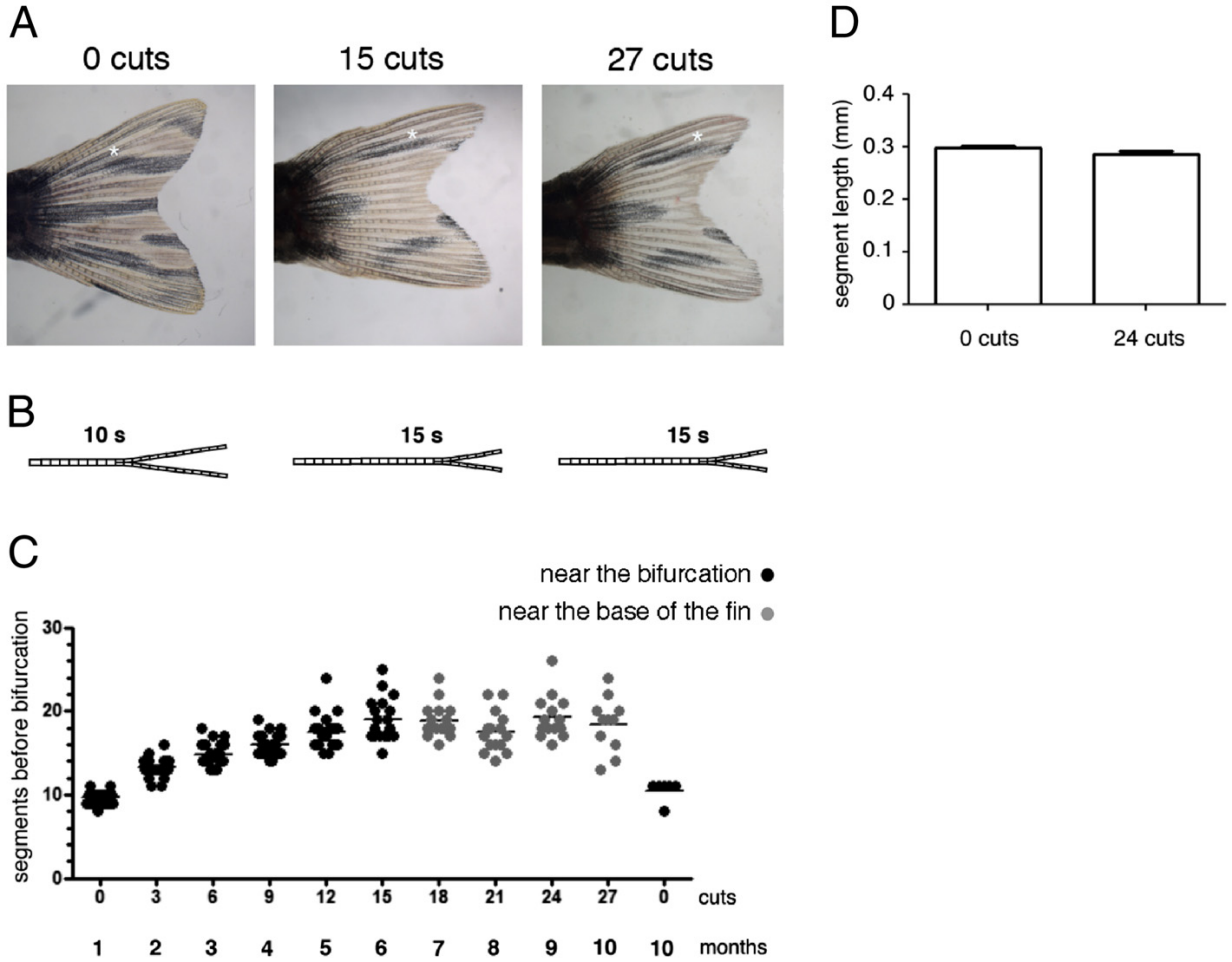
**The bifurcation position is distalized with repeated amputations. (A)** The same caudal fin before amputation and after 15 and 27 amputations. **(B)** Schematic representation of the bifurcation distalization with the repeated amputations. **(C)** Number of segments formed in the 3^rd^ dorsal ray between the base of the fin and the bifurcation after consecutive amputations. The caudal fin was subjected to three amputations every month and this was repeated 10 times. During the first 15 amputations, the third consecutive amputation (the last before allowing the fin to completely regenerate) was done three segments below the most proximal bony ray bifurcation (near the bifurcation). In the next 12 cuts, the third consecutive amputation was done 4 segments distally from the base of the fin (near the base of the fin). **(D)** 3^rd^ dorsal ray segment length before any amputation and after 24 amputations. Asterisk marks the bifurcation position. The fins were allowed to regenerate for 4 weeks between each round of three consecutive amputations.

Although the regenerative capacity was not affected [7], we detected an alteration in the original pattern of pigment cells and a distal shift in the position of the bony ray bifurcations in the regenerated caudal fins (Figure 1A, B). 

We quantified the number of segments formed between the base of the fin and the 3^rd^ dorsal ray bifurcation in the regenerated fin in order to determine the proximal-distal position of the bifurcation after each set of consecutive amputations. The 3^rd^ dorsal bony ray was used as a reference because this bifurcated ray localizes to the region of the fin containing the longest bony rays, thus providing a better proximal-distal morphological reference.

We observed that, after each set of amputations during the first 6 months, there was an increase in the number of segments formed between the base of the fin and the 3^rd^ dorsal ray bifurcation. This reveals that the position of the bifurcations was progressively shifted distally when compared to its position before amputation (Figure 1C - near the bifurcation). In the following 4 months, the number of segments formed between the base of the fin and the 3^rd^ dorsal ray bifurcation was maintained, showing that the proximal-distal position of the bifurcations was unaltered (Figure 1C – near the base of the fin). The distalization of bifurcation was not due to an increase in the number or length of segments with ageing during this 10-month experiment (Figure 1C, D).

These results show that the bifurcation position is distalized with repeated amputations.

### The bifurcation position is only shifted distally when the amputations are performed repeatedly near the bifurcations

One possibility to explain the maintenance of the proximal-distal position of the bifurcation observed in the last 4 months of our experimental setting could be that the distalization of the bifurcation reached its maximum limit after 6 months of consecutive amputations. Another possibility could be that the increased amputation distance from the bifurcation place would decrease the possible influence of an amputation in the bifurcation position after regeneration.

**Figure 2:**
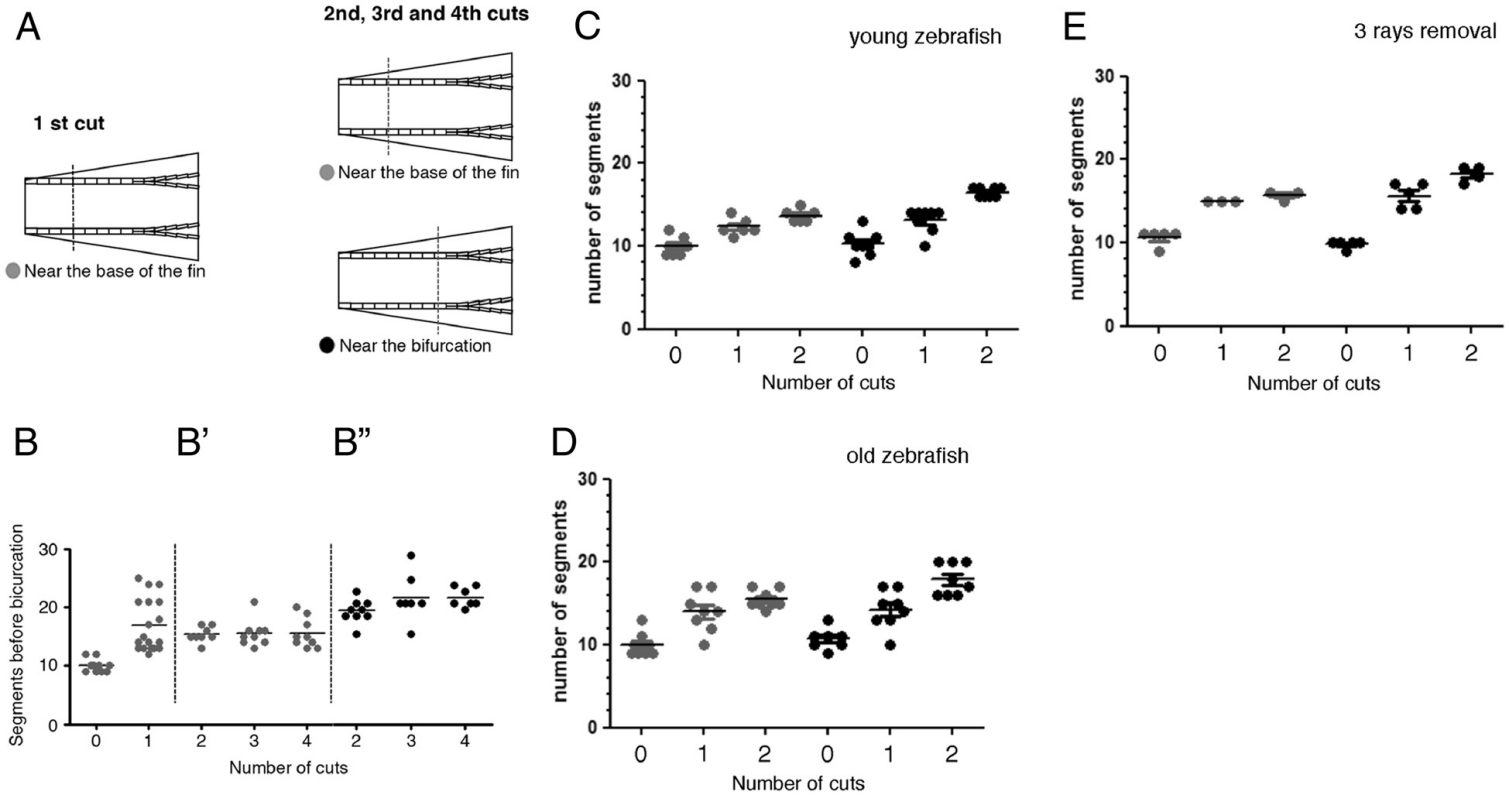
**The distalization of the bifurcation is dependent on the proximal-distal level of amputation. (A)** After a first amputation performed at 4 segments from the base of the fin, the fish were divided into two groups. One group was amputated a second, third and fourth time at 4 segments from the base of the fin (near the base of the fin) and the second group was amputated at one segment below the most proximal bifurcation (near the bifurcation). **(B)** Number of segments formed in the 3^rd^ dorsal ray between the base of the fin and the bifurcation after consecutive amputations performed always at 4 segments from the base of the fin **(B’)** and after a first amputation performed at 4 segments from the base of the fin followed by a second, third and fourth amputations near the bifurcation **(B”)**. Number of segments formed between the base of the fin and the 3^rd^ dorsal ray bifurcation after consecutive amputations performed at 4 segments from the base of the fin and at one segment below the most proximal bifurcation in 6 month old zebrafish **(C)** and in 1 year and 6 month of age zebrafish **(D)**. **(E)** Number of segments formed between the base of the fin and the 4^th^ dorsal ray after the removal of the 3^rd^, 4^th^ and 5^th^ bony rays at 4 segments from the base of the fin and at one segment below the most proximal bifurcation. Fins were allowed to completely regenerate for 2 weeks before the following amputation.

To distinguish between these two possibilities, we designed a more controlled amputation protocol (Figure 2A). We performed a first amputation at 4 segments from the base of the fin (near the base of the fin) in 20 adult zebrafish and allowed the fin to completely regenerate. The second, third and fourth amputations were performed at 4 segments from the base of the fin (near the base of the fin) in 10 of the animals and, in the remaining 10, the second, third and forth amputations were performed at 1 segment below the most proximal bifurcation (near the bifurcation).

Upon a first amputation near the base of the fin, the bifurcation was immediately distalized when compared to its position in the uncut fin (Figure 2B). Following the second, third and fourth amputations, the bifurcation position was maintained in the regenerated fin when the amputations were done near the base of the fin (compare Figure 2B with B’) while it was progressively distalized when the amputations were done near the bifurcation (compare Figure 2B with B”). The same modulation of the bifurcation position upon amputation of the caudal fin at different proximal-distal levels was observed in younger and older zebrafish (Figure 2C, D).

These data show that repeated amputations performed at a long distance from the bifurcation (i.e. at 4 segments from the base of the fin) do not change its proximal-distal position in the regenerated fin, while consecutive amputations near the bifurcation induce a positional reset and progressively shift its position distally.

### The bifurcation position is modulated by neighbouring regenerating tissues

We addressed whether the influence of the amputation level in the bifurcation position is the result of a global response of the entire fin or a local response of the tissues surrounding each amputated ray.

It was previously suggested that the inter-ray tissue is necessary for the bifurcation formation [8]. In this study, the authors show that upon single ray ablation, the ability of the ray to bifurcate depends on the presence of regenerating adjacent inter-ray tissue. Thus, in order to address whether the bifurcation position after amputations at different proximal-distal levels is modulated by local signals, we amputated the 3^rd^, 4^th^ and 5^th^ rays. We analysed the bifurcation of the 4^th^ ray, which had the influence of the neighbouring 3^rd^ and 5^th^ ray blastemas and regenerating inter-rays. We observed that the bifurcation position is only further distalized when the second amputation is performed at 1 segment below the bifurcation (near the bifurcation) (Figure 2E). Therefore, these data suggest that the modulation of the bifurcation position is influenced by a local signal, most likely present in the surrounding regenerating inter-ray, although we cannot exclude the contribution of the adjacent blastemas.

### The induced *shh* expression pattern is independent of the place of amputation

Sonic hedgehog (Shh) is a strong candidate to be the trigger of bifurcation formation in the zebrafish caudal fin [9]. It was reported that at 2 and 3 days-post-amputation (dpa), a strong single domain of *shh* expression is detected at the level of amputation on the top of each hemiray. By 4 dpa, this *shh* single domain starts to split into two groups of cells located laterally in the proximal region of the basal wound epidermal layer. This shift in *shh* expression from one to two domains was proposed to correlate with the formation of a bifurcation during fin regeneration [9]. Thus, we wanted to determine how this dynamic expression pattern of *shh* is modulated by the amputation place.

**Figure 3:**
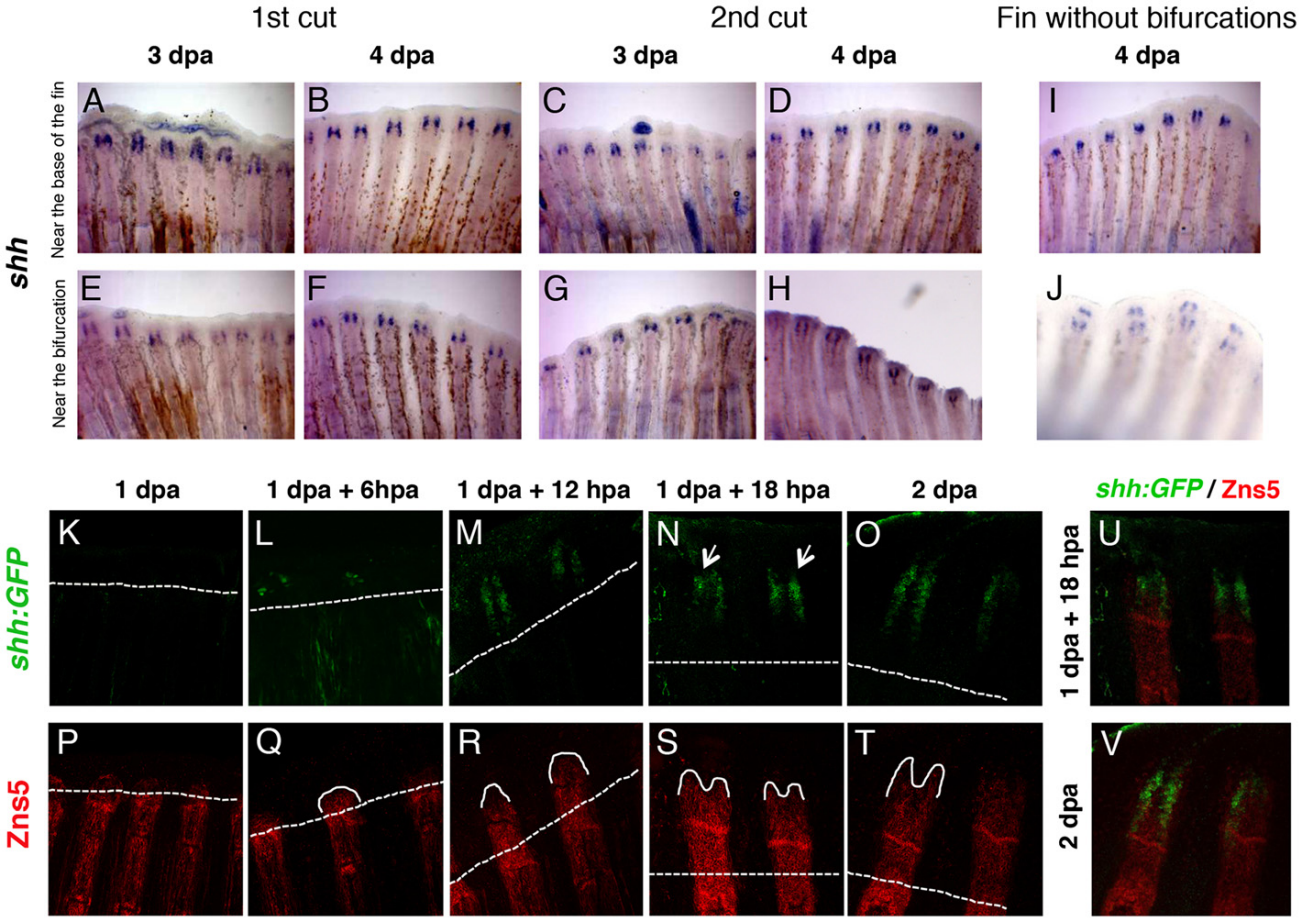
**The expression pattern of*shh*during regeneration does not change with the proximal-distal level or the number of amputations.(A-D)** Caudal fins were amputated at 4 segments from the base of the fin (near the base of the fin) once or twice and *shh* expression was determined at 3 or 4 days following the amputation. **(E-H)** Caudal fins were amputated at 1 segment below the bifurcation (near the bifurcation) once or twice and *shh* expression was determined at 3 or 4 days following the amputation. **(I)** Caudal fins with no bifurcations were amputated near the base of the fin and *shh* expression was examined at 4 days following the amputation. **(J)** Top view of the caudal fin shown in I. **(K-V)** Caudal fins of 2.2*shh*:*gfp:*ABC#15 transgenic fish were amputated near the bifurcation and analyzed at different time-points after amputation by a double immunostaining with anti-GFP (green) **(K-O)** and anti-Zns5 (red) **(P-T)** antibodies. **(U,V)** merge of 1 dpa + 18 hpa **(U)** and 2 dpa **(V)**. Dashed line represents amputation plane. hpa: hours-post-amputation; dpa: days-post-amputation. Between the first and the second amputation, fins were allowed to regenerate for 2 weeks.

For this purpose, we performed two rounds of amputations in two different places, at 1 segment proximal to the bifurcation (near the bifurcation) or at 4 segments from the base of the fin (near the base of the fin) since we have observed that the second amputation in different proximal-distal places modulates the bifurcation position. We then analysed the dynamics of *shh* expression at 3 and 4 dpa by in situ hybridization. Interestingly, we observed that, independently on the number and places of the amputations, *shh* was consistently expressed in two separate cellular domains already at 3 dpa (Figure 3A-H). These results show that *shh* expression is not modulated by the amputation place. Moreover, at 4 dpa, in caudal fins that did not possess any bifurcations after being subjected to several distal amputations, *shh* expression was localized in two groups of cells located laterally in the proximal region of the basal wound epidermal layer (Figure 3I, J). This strongly suggests that Shh expression is not sufficient to trigger the formation of bifurcations.

We used confocal microscopy to analyse the dynamics of *shh* expression in the regenerating fin using the zebrafish transgenic expressing GFP under the control of the *shh* promoter (2.2*shh*:*GFP:*ABC#15) [10]. We performed one amputation, at 1 segment proximal to the bifurcation (near the bifurcation) and analysed the expression of *shh:GFP,* every 6 hour from 1 to 2 dpa. The time course analysis revealed that the establishment of *shh:GFP* expression pattern during regeneration is around 1 dpa + 12 hpa. *shh:GFP* expression, is absent at 1 dpa (Figure 3K) and, in a few cases, can be detected at 1 dpa + 6 hpa, in a small number of cells, in one or both sides of the regenerating hemiray (Figure 3L). From its onset of expression (at 1 dpa + 12 hpa) until 2 dpa, *shh:GFP* is always present with the same pattern of expression, namely two separate groups of cells (Figure 3M-O). By performing a higher cellular resolution analysis, we never observed a transition in *shh:GFP* expression from one to two domains during fin regeneration. These results provide additional support to the conclusion that Shh may not be the instructor to form the bifurcation.

In addition, it has also been proposed that Shh plays a role in the patterning and/or differentiation of osteoblasts within the blastema during fin regeneration [6]. In order to determine whether there is a correlation between the restriction of *shh* expression in two epidermal domains and the dynamics of bone formation during regeneration, we performed a Zns5 (osteoblast marker) immunostaining time-course analysis (every 6 hours from 1 to 2 dpa) in the 2.2*shh*:*GFP:*ABC#15 transgenic fish (Figure 3P-T). Interestingly, we observed that soon after the onset of *shh:GFP* reporter expression, the growing bone alters the shape of its tip from a cone to a “V” shape (compare Figure 3R with S). This shows that, Zns5+ cells cease to be localized in the middle of the differentiating bone and are aligned close to the basal layer of the epidermis where *shh* mRNA is produced (Figure 3N, O, S-V). Interestingly, we have also observed that *shh:GFP* expression domains can be irregular in pattern and differs in the number of *shh:GFP* positive cells in each individual blastema of the same fin (Figure 3N - arrows). Consequently, the visibility of *shh* separation in two cellular domains depends on the regenerating ray and blastema shape. Similarly, irregularities in the shape are also visible in the spatial organization of Zns5+ cells in the regenerating tip of each ray (Figure 3P-T).

Altogether, these results suggest that *shh* expression in two separate domains in the basal layer of the epidermis is not instructing the proximal-distal position of the bony ray bifurcation, but could have an important role in bone formation and growth possibly through osteoblasts alignment by attracting them to the region where Shh is being produced.

### Fgf does not play a role in the determination of the bone bifurcation position

The levels of Fgf signalling activation vary according to the proximal-distal place of amputation. Upon amputation, the expression levels of Fgf downstream targets such as *mkp3*, *sef* and *spry4* are higher following a proximal amputation when compared to a distal amputation [11]. This suggests the existence of positional memory possibly mediated through Fgf signalling.

In order to investigate whether Fgf signalling determines the proximal-distal position of the bifurcation in the regenerated fin, we made use of the *hsp70:dn-fgfr1* transgenic zebrafish [11]. This transgenic contains a dominant-negative fgfr1-EGFP fusion gene (dnfgfr1-EGFP) driven by a heat-inducible zebrafish hsp70 promoter. It was previously demonstrated that this construct attenuates Fgf signalling during fin regeneration in a dose dependent manner. Upon heat-shock, the regeneration growth rate is affected. This phenotype is highly sensitive to 1°C temperature increments [11].

**Figure 4:**
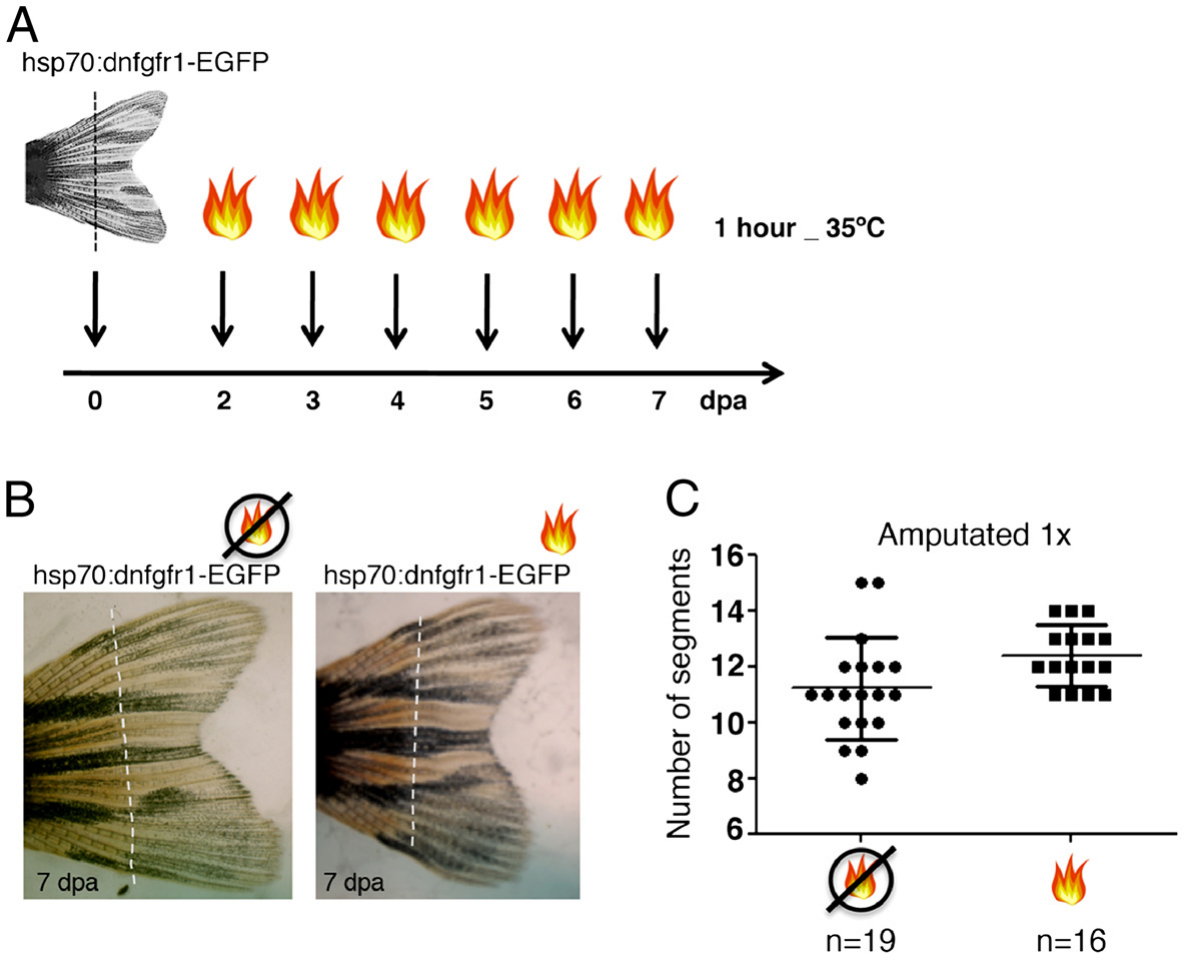
**Fgf signalling does not play a role in the determination of the proximal-distal level where the bifurcation will form. (A)** Heat-shock protocol following an amputation. Transgenic *hsp70:dn-fgfr1* fish were amputated at 1 segment proximal to the bifurcation (near the bifurcation) and heat-shocked at 35°C for 1 hour, during 6 days, starting at day 2 post amputation. **(B)** Picture of a 7 days-post-amputation regenerated caudal fin of *hsp70:dn-fgfr1* transgenic fish with or without the heat-shock protocol. **(C)** Number of segments formed in the 3^rd^ dorsal ray between the base of the fin and the bifurcation after one amputation near the bifurcation in heat-shocked and non-heat-shocked siblings. Dashed line represents amputation plane.

The *hsp70:dnfgfr1-EGFP* transgenic zebrafish were amputated once, at 1 segment proximal to the bifurcation (near the bifurcation) and Fgf signalling was partially inhibited by heat-shocking at 35°C for 1 hour daily, starting at day 2 until day 7 post-amputation (Figure 4A). The time-window of this protocol was designed to target the regenerative outgrowth phase (when the bifurcations are most probably signalled to form) at a temperature that does not block regeneration. The induction of *dnfgfr1* upon heat-shock was confirmed by the detection of GFP in the regenerating fins. The regenerated caudal fins after this protocol presented the bifurcation place in the same proximal-distal position as the amputated non heat-shocked siblings, as analysed by counting the number of segments formed between the base of the fin and the 3^rd^ dorsal bony ray bifurcation (Figure 4B, C). Other protocols of attenuation of Fgf signalling were tested by heat-shocking at different temperatures, durations or time-points of regeneration. However, none of the protocols tested affected the bifurcation position (i.e. the number of segments formed between the base of the fin and the bony ray bifurcation in the regenerated caudal fin) (Additional file 1: Figure S1). These results suggest that Fgf signalling is not involved in the determination of the bony ray bifurcation position during caudal fin regeneration, although they do not exclude its involvement in the size control or in the regulation of proliferation levels upon amputation at different proximal-distal levels.

## Discussion

The existence of positional memory during appendage regenerative outgrowth comes largely from the work performed in amphibians. Blastema cells of an amphibian limb inherit a memory of their initial position and specify the proximal boundary of the regenerate. This boundary will prevent blastema cells to form structures proximal to their level of origin [12]. The proximal-distal axis of a regenerating amphibian limb can be viewed as a series of such boundaries since the limb always regenerates the missing elements with the correct patterning and identity upon amputation at different levels [12]. In the amphibian limb, positional information is easy to recognize since there is a clear proximal-distal succession of different bone elements.

In zebrafish, evidence for positional information comes from the fact that a caudal fin will regenerate a similarly shaped fin after being amputated at different proximal-distal levels [13]. Moreover, a proximal amputation presents a greater growth rate, which correlates to the higher number of proliferating cells detected when compared to a distal amputation [11]. However, how different proximal-distal amputation places will impact on the positional information of the caudal fin bony rays has not been studied probably because bone landmarks are scarce in the caudal fin. Since most bony rays bifurcate in a defined distal position, this is the only morphological feature that can be used as a reference of positional memory. Even though several reference points would be ideal for the study of positional information, the number of segments before bifurcation is an objective reference in the proximal-distal axis of the fin.

Using bony ray bifurcations as landmarks, we were able to show that, in contrast to what happens in amphibians, the amputation place influences the bony ray bifurcation position. Repeated amputations performed near the bifurcation will progressively induce a distal shift, changing the original position of the bifurcation and resetting its positional information. We have also observed that the distalization of the bifurcation is independent of the proximal-distal place of amputation before the second amputation. This is likely explained by the short distance between the amputation plane and the bifurcation observed before the first amputation even when the amputation is done near the base of the fin. This distance is increased after the first amputation, resulting in the elimination of the influence of the amputation in the bifurcation position when the amputation is done near the base of the fin. Our data is consistent with the previously reported increase in the number of segments formed before a bifurcation when an amputation is done 2–3 segments below the bifurcation [14]. This means that a certain number of segments will need to form/differentiate before a bifurcation is signalled to form. Moreover, we show that the bifurcation position is modulated by neighbouring regenerating tissues. Thus, it is possible that upon amputation, the regenerating surrounding tissues, namely the blastemas and inter-rays will inhibit the signal(s) responsible to initiate the cell and molecular mechanisms of a bifurcation and consequently delay its formation.

Previous reports have shown that preceding the formation of a bony ray bifurcation during caudal fin regeneration, *shh* splits in two its single domain of expression in the basal layer of the epidermis [9]. This indicates that Shh is a good candidate to signal the formation of a bifurcation [6,9]. Therefore, we hypothesized that the place of amputation could modulate the dynamics of *shh* expression and therefore the proximal-distal level at which a bifurcation will form. However, we observed that the dynamics of *shh* expression do not change with different proximal-distal amputation places, being always expressed in two separate groups of cells in the basal layer of the epidermis. This new pattern of *shh* expression that we have uncovered was possible to determine due to a high cellular resolution analysis that we performed. Furthermore, in caudal fins without any bifurcations, after being submitted to several distal amputations, the expression of *shh* is always observed in two separate domains. Thus, our results suggest that Shh cannot be the instructing signal responsible for positioning the bony ray bifurcation in a regenerating caudal fin.

We propose however that Shh, may be important for the formation of bone at the right place, acting has an attractor of bone progenitors aligning them, directing bone growth and possibly controlling the width of the bony rays in the regenerating fin. This conclusion is based on our time-course analysis of Zns5-expressing cells in the context of a *shh* reporter line. This analysis revealed that soon after the detection of *shh* expression, osteoblasts in the bone growing tip start to align close to the basal layer of the epidermis next to *shh*-expressing cells. This interpretation is consistent with previous findings that proposed that Shh might play a role in the osteoblasts patterning during fin regeneration [6].

It has been previously demonstrated that Fgf targets show higher expression levels in proximal regenerates when compared to distal ones. This suggests the existence of an Fgf gradient in the regenerating fin, which indicates that Fgf signalling might be implicated in the regulation of positional memory during fin regeneration. [11]. Moreover, Fgf signalling is required for the expression of the homeobox-containing gene, *msxb*[15] which, accordingly to an earlier report, is differentially expressed along the proximal-distal axis of the fin [16]. Thus, Fgf signalling would be a good candidate to modulate the position of the bony ray bifurcation.

In order to address a potential role of Fgf signalling in determining the bifurcation position, we made use of a heat-shock inducible transgenic to attenuate Fgf signalling in a time controlled manner. All the different protocols used to transiently attenuate Fgf signalling did not alter the position of the bony ray bifurcation when compared to the controls with unaffected Fgf signalling levels. This indicates that Fgf signalling is not likely to be the factor controlling the formation of a bony ray bifurcation in the zebrafish regenerating caudal fin.

Retinoic acid (RA) is an additional strong candidate to be involved in the regulation of positional information. Evidence for this comes from relevant work in the amphibian limb where a gradient of RA and of the cell surface protein CD59 was shown, with higher levels in more proximal blastemas when compared to the distal ones [17,18]. In addition, treatment with RA stimulates regeneration of proximal structures in a concentration-dependent fashion [19,20] by increasing the levels of CD59 [18].

In contrast, the role of RA in the positional memory of the regenerating zebrafish caudal fin remains poorly understood. It has been proposed that RA treatment distalizes the bifurcation point due to the fusion of fin rays [13,14]. It is not clear though, whether this is caused by a proximalization of the regenerating tissue, by the downregulation of *shh* following RA treatment [9], which leads to defects in bone formation/patterning [6] or even toxicity [21]. Therefore, the role of RA in the positional memory of the regenerating fin should be further investigated.

Positional memory is a complex process that is likely to involve local interactions between different cell types and domains and multiple signalling pathways. In fact, a crosstalk between blastema, distal epidermis and inter-ray tissue was demonstrated to be essential to signal the formation of a bifurcation in regenerating zebrafish fins [8]. More recently, a mathematical model proposes that the regeneration of a fin with the correct shape and pattern requires the interplay of three morphogens [22]. Future studies will be essential to uncover the signals that give positional information to the regenerating fin/intact fin tissue.

## Conclusions

Our results challenge the idea of the existence of a simple mechanism of positional information in the regenerating zebrafish caudal fin. In fact, the place of the bony ray bifurcation is progressively shifted to a more distal position when repeated amputations are performed near the bifurcation. In addition, we found that this modulation is regulated by the immediate surrounding tissues (inter-rays and blastemas). In search of the signal involved in the modulation of the bifurcation position, we analysed in detail the expression of *shh*, which has been pointed has a strong candidate in triggering the bifurcation formation. With our high-resolution analysis, we describe a different progression of *shh* expression during zebrafish caudal fin regeneration, which does not correlate with the determination of the bifurcation place. Instead, our results suggest that this morphogen is involved in the alignment of the bone forming cells during the regeneration process.

## Methods

### Ethics statement

All experiments involving animals were approved by the Animal User and Ethical Committees at Instituto de Medicina Molecular, according with directives from Direcção Geral Veterinária (PORT 1005/92).

### Zebrafish lines, maintenance and surgery

The following zebrafish strains were used in this study: wild-type AB strain (from ZIRC), Tg(*hsp70:dn-fgfr1*pd1 strain [11] and 2.2*shh*:*gfp:*ABC#15 [10]. 6–24 months of age zebrafish were anaesthetized in 0.1% tricaine (Sigma- Aldrich), and caudal-fin amputations were performed with razor blades. Animals were allowed to regenerate for various times in water kept at 30-33°C, except the Tg(*hsp70:dn-fgfr1*pd1 strain that was keep at 28.5°C.

### Adult heat induction experiments

A heated incubator was used to maintain the water of breeding boxes warmed to the heat-shock temperature of 35°C or 34°C, 36°C and 38°C (for the experiments in Additional file 1: Figure S1). To give the heat-shock, zebrafish were transferred from a temperature of 28,5°C to the breeding boxes with heated water in the incubator.

### In situ hybridization

The antisense *shh* RNA probe was synthesized with a digoxigenin labelling kit (Promega) and as previously described by Henrique et al. (1995). The plasmid containing *shh* cDNA was kindly provided by David Wilkinson’s lab. In situ hybridization of zebrafish fins was performed as follows. Fin regenerates were fixed overnight at 4°C in 4% paraformaldehyde (PFA) in phosphate-buffered saline (PBS) and transferred to ethanol at room temperature (RT) and stored at −20°C, at least one overnight. Fins were rehydrated stepwise through ethanol in PBS-0,1% Triton (PBT) and washed in two changes of PBT for 10 min. A solution of 6% of H_2_O_2_ in PBT was used during 30 min to inactivate endogenous peroxidases, followed by two washes for 5 min in PBT. Proteinase K (10 mg/ml) digestion was performed for 15 min and then stopped by washing with a glycine solution (2 mg/ml in PBT). After two washes for 5 min in PBT, fins were refixed with 3.7% Formaldehyde solution, 0.2% Glutaraldehyde in PBT for 20 min followed by another two PBT brief washes. Pre-hybridization was allowed for ≥1 h at 70°C, in hybridization solution (Hyb solution) containing: 60% formamide, 5x SSC (20x pH 6.0), 500 mg/ml tRNA, 0,1% Tween20 (10%), 50 mg/ml heparin, in miliQ H_2_O. Fins were then hybridized in Hyb solution, containing 5 ml/ml digoxigenin-labeled RNA probe, overnight at 70°C. Unhybridized probe was removed using washing solutions I and II (washing solution I: Formamide 50%, 1× SCC, 0.1% Tween 20; washing solution II: 50% Wash I, 50% TBST) at 70°C for 15–30 min (wash I: 2 × 15 min + 2 × 30 min; wash II: 2 × 20 min). After this fins were washed with TRIS-buffered saline in 0,1% Tween 20 (TBST), incubated in a blocking solution (10% sheep serum in TBST) at RT for ≥1 h and incubated with anti-digoxigenin antibody coupled to alkaline phosphatase Fab fragment (Roche), 1:2500 in blocking solution (10% goat serum in TBST), overnight at 4°C. The excess of anti-digoxigenin antibody was removed with at least four TBST washes for 15 min. For the alkaline phosphatase reaction, fins were first washed in reaction buffer NTMT (5 M NaCl, 1 M Tris HCl pH 9.5, 1 M MgCl_2_, Tween20, H_2_O MQ) for 5 min followed by two washes for 10 min. The staining signal was developed with the staining reaction containing 2 μL/mL NBT and 3.5 μL/mL BCIP (Roche).

### Immunohistochemistry

The fins were fixed in a solution of 80% methanol, 20% DMSO (Sigma) overnight at 4°C, rehydrated in a methanol-PBS series, permeabilised with acetone at −20°C for 20 min, followed by two washes in PBS. An additional permeabilisation step was done with a PBST 0.5% solution (PBS with 0.5% Triton X-100) for 30 min. Fins were then washed several times with PBS, blocked in PBS with 10% foetal bovine serum (FBS) and incubated with the primary monoclonal antibody anti-Zns5 (dilution 1:250) (ZIRC 011604) to mark osteoblasts and anti-GFP antibody (dilution 1:400) (Abcam) overnight at 4°C. After several washes in PBS fins were incubated with the secondary antibody overnight at 4°C and then mounted for analysis.

### Microscopy

Images of in situ hybridisations were obtained with a Leica Z6APO stereomicroscope equipped with a Leica DFC490 digital camera. Images of immunostainings were obtained on a Zeiss LSM 510 META confocal microscope. Captured Z stacks were analysed using ImageJ software.

## Authors’ contributions

ASS designed the experiments, performed all the experiments and wrote the manuscript. SS made the bone growth shape observation that was further investigated in Figure 3. AJ designed the experiments. LS designed the experiments and wrote the manuscript. All authors read and approved the final manuscript.

## Supplementary Material

Additional file 1 Figure S1 Fgf signalling does not seem to play a role in the determination of the proximal-distal position of the bifurcation. Transgenic *hsp70:dn-fgfr1* fish were amputated 1 segment proximal to the bifurcation and heat-shocked at: 35°C for 1 hour, every other day, from day 2 post amputation until day 8 post amputation; 36°C for 1 hour daily, during 3 days, starting at day 2 post amputation; 34°C permanently, from the time of amputation until the accomplishment of a complete regeneration; once at 38°C for 1 hour at 2 dpa. The number of segments formed in the 3^rd^ dorsal ray between the base of the fin and the bifurcation in the heat shocked zebrafish were counted and compared to the non-heat-shocked siblings **(A)** or to the heat-shocked siblings, negative for *hsp70:dn-fgfr1* insertion **(B)**. dpa: days-post-amputation. Click here for file
