# Supplementary figures and images for: An amputation resets positional information to a proximal identity in the regenerating zebrafish caudal fin
Source: BMC Dev Biol. 2012 Aug 25;12:24. doi: 10.1186/1471-213X-12-24 (PMC3484062; doi:10.1186/1471-213X-12-24)

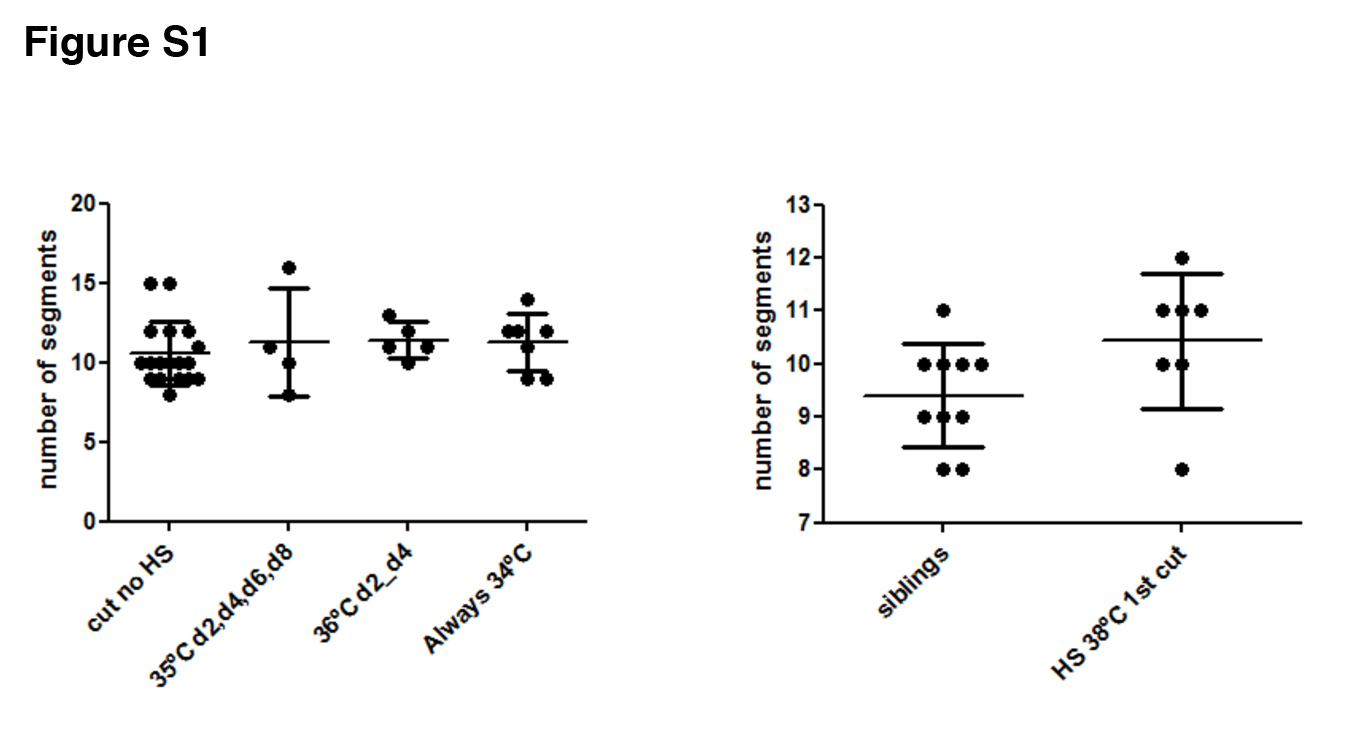

Supplement: Additional file 1 Figure S1 — Fgf signalling does not seem to play a role in the determination of the proximal-distal position of the bifurcation. Transgenic hsp70:dn-fgfr1 fish were amputated 1 segment proximal to the bifurcation and heat-shocked at: 35°C for 1 hour, every other day, from day 2 post amputation until day 8 post amputation; 36°C for 1 hour daily, during 3 days, starting at day 2 post amputation; 34°C permanently, from the time of amputation until the accomplishment of a complete regeneration; once at 38°C for 1 hour at 2 dpa. The number of segments formed in the 3rd dorsal ray between the base of the fin and the bifurcation in the heat shocked zebrafish were counted and compared to the non-heat-shocked siblings (A) or to the heat-shocked siblings, negative for hsp70:dn-fgfr1 insertion (B). dpa: days-post-amputation. [file 1471-213X-12-24-S1.jpeg]
